# Predictors and outcomes of recurrent retroperitoneal liposarcoma: new insights into its recurrence patterns

**DOI:** 10.1186/s12885-023-11586-8

**Published:** 2023-11-08

**Authors:** Huan Deng, Jingwang Gao, Xingming Xu, Guibin Liu, Liqiang Song, Yisheng Pan, Bo Wei

**Affiliations:** 1 Department of Gastrointestinal Surgery, Peking University First Hospital, Beijing, 100034 China; 2 Department of General Surgery, the First Medical Center, Chinese People’s Liberation Army General Hospital, Beijing, 100853 China

**Keywords:** Retroperitoneal liposarcoma, Recurrence pattern, Prognosis, Distant recurrence, Local recurrence

## Abstract

**Background:**

The clinical profiles of recurrent retroperitoneal liposarcoma (RLS) need to be explored. The recurrence patterns of RLS are controversial and ambiguous.

**Methods:**

A total of 138 patients with recurrent RLS were finally recruited in the study. The analysis of overall survival (OS) and recurrence-free survival (RFS) was performed by Kaplan‒Meier analysis. To identify independent prognostic factors, all significant variables on univariate Cox regression analysis (*P* ≤ 0.05) were subjected to multivariate Cox regression analysis. The corresponding nomogram model was further built to predict the survival status of patients.

**Results:**

Among patients, the 1-, 3-, and 5-year OS rates were 70.7%, 35.9% and 30.9%, respectively. The 1-, 3- and 5-year RFS rates of the 55 patients who underwent R0 resection were 76.1%, 50.8% and 34.4%, respectively. The multivariate analysis revealed that resection method, tumor size, status of pathological differentiation, pathological subtypes and recurrence pattern were independent risk factors for OS or RFS. Patients with distant recurrence (DR) pattern usually had multifocal tumors (90.5% vs. 74.7%, *P* < 0.05); they were prone to experience changes of pathological differentiation (69.9% vs. 33.3%, *P* < 0.05) and had a better prognosis than those with local recurrence (LR) pattern. R0 resection and combined organ resection favored the survival of patients with DR pattern in some cases.

**Conclusions:**

Patients with DR pattern had better prognosis, and they may benefit more from aggressive combined resection than those with LR pattern. Classifying the recurrence patterns of RLS provides guidance for individualized clinical management of recurrent RLS.

**Supplementary Information:**

The online version contains supplementary material available at 10.1186/s12885-023-11586-8.

## Background

Retroperitoneal liposarcoma (RLS) is a rare malignant tumor arising in the retroperitoneum and is the most common type of retroperitoneal sarcoma. RLS accounts for 0.07% to 0.2% of all tumors and approximately 12% and 40% of all liposarcomas [1]. This tumor can be further divided into 4 subtypes: well-differentiated liposarcoma (WDL), dedifferentiated liposarcoma (DDL), myxoid cell liposarcoma (MLS), and pleomorphic liposarcoma (PLS) [2]. WDL and DDL are the major subtypes of RLS [3]. The prognosis of RLS is correlated with the pathological type and resection method. Previous studies have proven that poor differentiation of RLS promotes local recurrence and distant metastasis [4]. Surgical resection is currently the main treatment for this disease, but those patients have a higher relapse propensity even after complete resection [5]. However, the resection margin and histological subtype remain the most important prognostic predictors for local recurrence and overall survival of RLS [3, 6, 7].

Numerous studies have demonstrated that combined resection of adjacent organs such as the kidney and gastrointestinal tissues in the abdomen will improve the local outcome [8]. However, the large tumor size and complicated anatomic structure of RLS limit the ability of the surgeon to achieve negative surgical margins [9]. Complete capsular resection and combined organ resection also rarely achieve radical cure of RLS, which is the surgical dilemma [10]. Previous studies revealed that RLS usually present as recurrence or metastasis patterns after surgical resection [11, 12]. However, there are some recurrent cases of cancer that are distinctly different from traditional recurrence [13]. As the frequency of recurrence increased and pathological differentiation changed, the tumor location may shift but not metastasize. These phenomena indicate the possibility of some different relapse mechanisms existing in tumorigenesis. We found that the phenomenon also exists in the recurrence of RLS. However, no reliable guidelines or studies have defined the subset of patients.

We analyzed the basic clinical and pathological characteristics of patients with recurrent RLS. We evaluated 138 cases of recurrent RLS in our department, proposing a novel relapse classification to explore the differences between LR and DR patterns in the retroperitoneum. This classification may provide evidence for individualized treatment of recurrent RLS.

## Materials and methods

### Patient selection

Cases of recurrent RLS were selected from the First Medical Center, Chinese People Liberation Army General Hospital from February 2000 to August 2017. All patients experienced recurrence and underwent at least twice surgeries in our hospital. The pathology was diagnosed and confirmed by experienced pathologists based on WHO (World Health Organization) criteria (WDL, DDL, MLS, PLS) [14]. Patients were excluded from the study if they experienced distant organ metastasis. Patients who received adjuvant radiotherapy or chemotherapy were also not enrolled in this study. The patients without complete medical records or follow-up data were excluded. Those patients with recurrence, who didn’t receive surgical treatment, were excluded in the study. The recurrence of RLS was confirmed based on radiological examinations, pathological examinations and surgical records. The extent of resection was discussed and confirmed by experienced radiologist, pathologist and surgeons in our hospital.

### Definitions

**Fig. 1:**
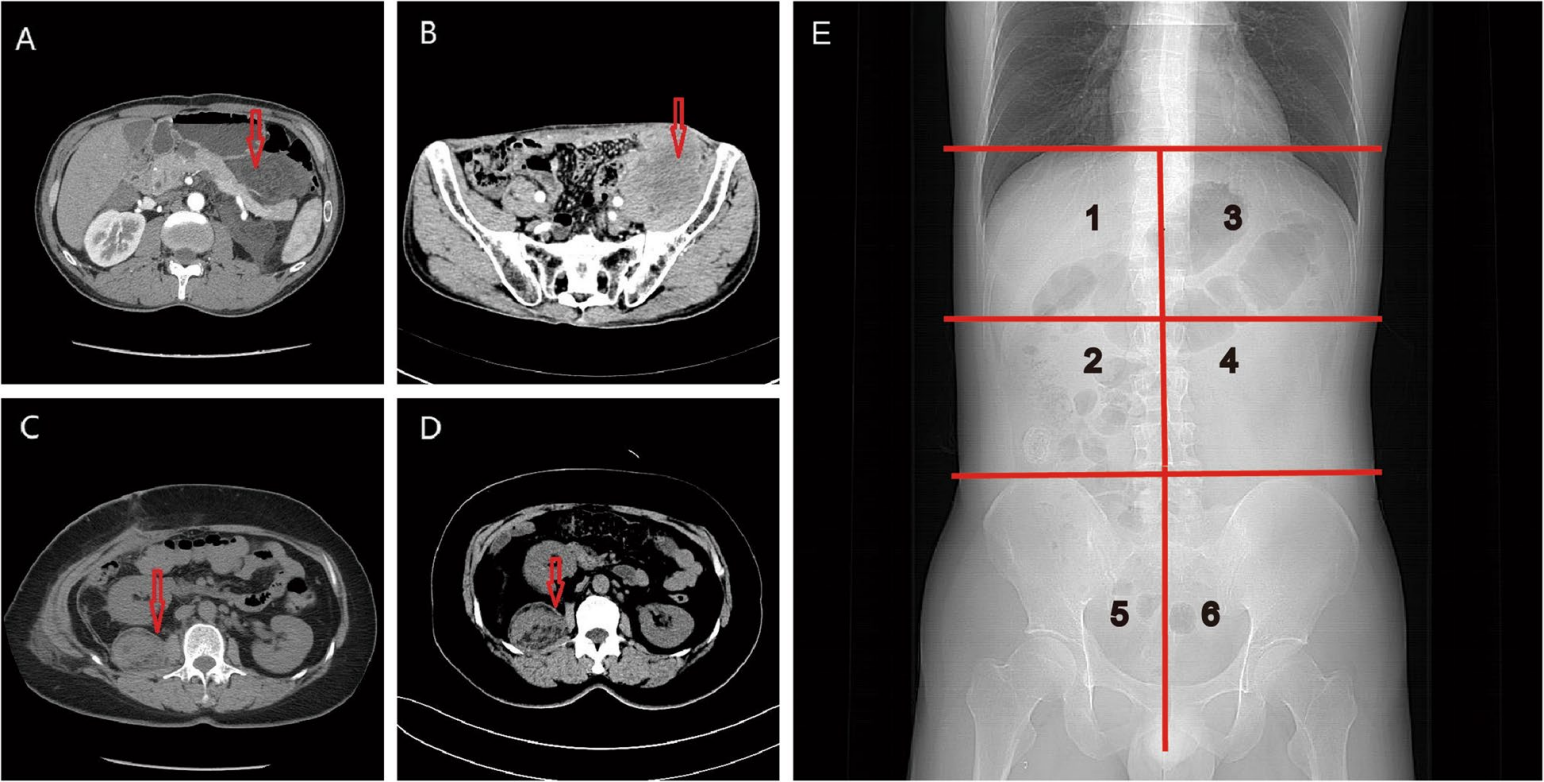
Two cases of recurrent RLS with different recurrence patterns and the schematical of retroperitoneal compartments in X-ray illustration. **A** Preoperative CT imaging of a patient with recurrent RLS adjacent to the spleen. **B** Preoperative CT imaging of the case with re-recurrence RLS in the left pelvic compartment. Images A and B shows two continuous recurrences of RLS in a patient with DR pattern. **C** Preoperative CT imaging of a patient with recurrent RLS in the right renal compartment. **D** Preoperative CT imaging of the case with re-recurrence RLS in the right renal compartment. Images C and D (Preoperative CT examinations) show two continuous recurrences of RLS in another patient with LR pattern. **E** Illustration of retroperitoneal compartments by X-ray. Six compartments were divided by three horizontal lines and a vertical line. Three horizontal lines represent the diaphragm level, L2 or renal (a, v) level, and pelvic brim level. The vertical line represents the midline of the spine. CT: Computerized tomography; RLS: retroperitoneal liposarcoma

Recurrent RLS was defined as tumors that relapse at least once from the initial diagnosis. Multifocal tumors were defined as the presence of two or more noncontiguous neoplasms. Local recurrence was defined as tumor relapse at the same anatomical compartment in the retroperitoneum. Distant recurrence was defined as tumor location altered to another compartment. The tumor location was evaluated combined preoperative radiological examinations and surgical dictations. To describe the tumor location and recurrence patterns of RLS, we showed the representative images of computerized tomography (CT) and schematical X-ray illustration of retroperitoneal compartments (Fig. 1A-E) [11].

Resection method was characterized as R0 (complete resection), R1 (microscopic tumor at margin) and R2 (palliative resection). The extent of R0/R1 resection was grossly complete, R2 resection was grossly incomplete [7]. Tumor growth rate (TGR) was defined as tumor size (the maximum dimension of the largest mass recorded on final pathological records) divided by the time from last resection to this recurrence diagnosed [3]. Overall survival (OS) referred to the time from surgical resection to the end of 5-year follow-up or death. Recurrence-free survival (RFS) was defined as the time from surgical resection to the onset of recurrence or death within 5 years [15].

### Statistical analyses

Categorical data are expressed as frequencies (percentages) and were compared using the chi-square or Fisher exact test. Continuous variables are expressed as the median (Q1-Q3). All variables significant on univariate Cox regression analysis (P ≤ 0.05) were subjected to multivariate Cox regression analysis. The Cox regression models were established by the survival coxph function of the R package. The nomogram model was built to predict the survival status of patients with recurrent RLS. The calibration of model was performed in training set (60%), validation set (40%) and external database. The external data from SEER (Surveillance, Epidemiology, and End Results) database were used for calibration model. Kaplan‒Meier curves were used to estimate the OS and RFS. The data were analyzed using IBM SPSS Statistics (Version 25.0) and GraphPad Prism (Version 8). A two-sided *P* value < 0.05 was considered statistically significant.

## Results

### Patient characteristics

**Fig. 2:**
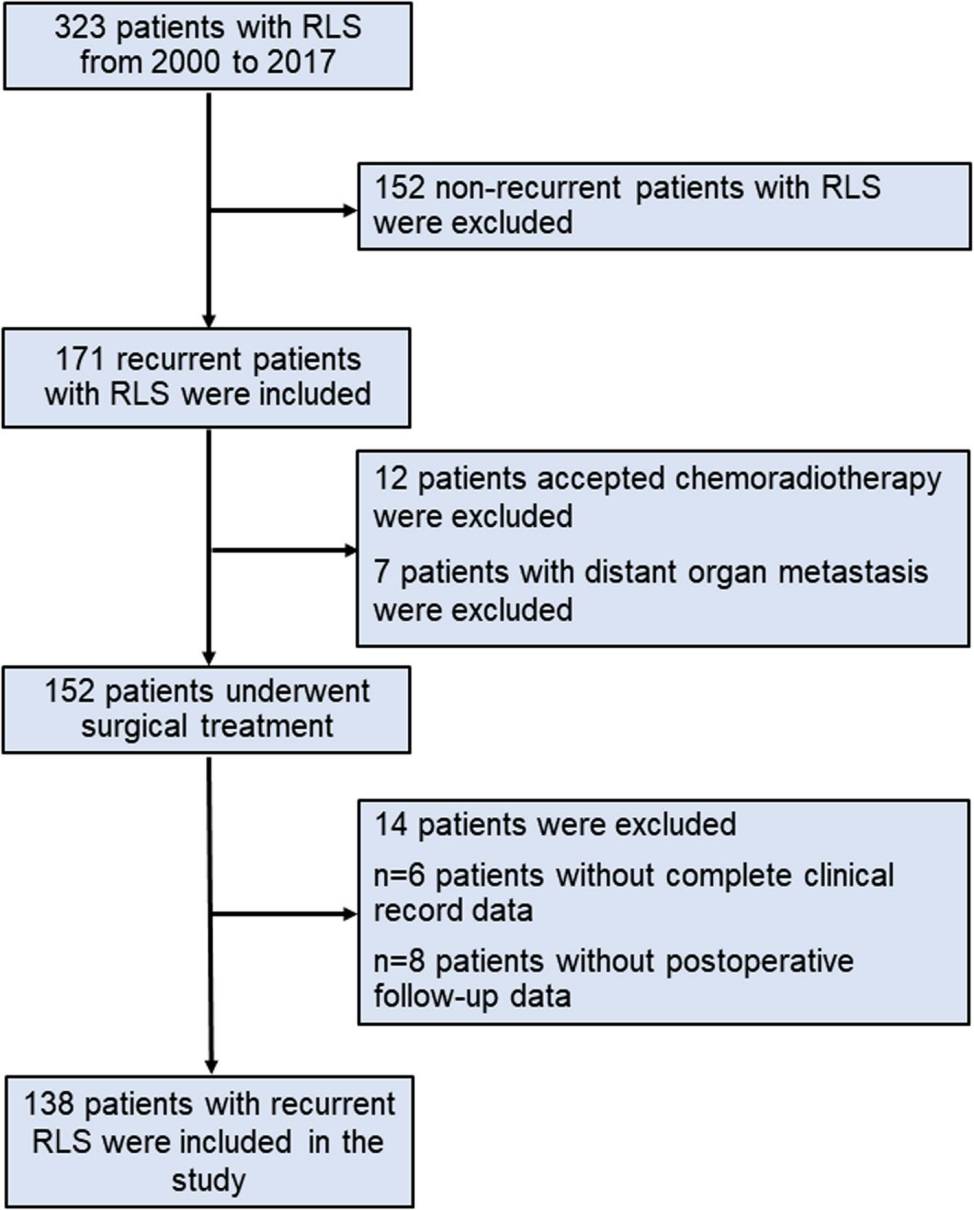
Patient flow diagram of recurrent RLS. RLS: retroperitoneal liposarcoma

A total of 138 patients with recurrent RLS were finally enrolled in the study (Fig. 2). Among them, the median age of the patients was 53 years, 77 patients were male and 61 patients were female. Forty-five percent of patients recurrence with DR pattern. Among all the patients, the median tumor size reached 18 cm, and 39.9% of patients achieved an R0 resection (Table 1). In this study, excess a half of patients experienced a change of pathological differentiation. The reciprocal changes between DDL and WDL were the dominant changes in our cohort (Supplementary Fig. 1). The median TGR of relapsed tumors was 1.28 cm/month. The distribution of clinical and pathological characteristics among patients is illustrated in Table 1.

**Table 1:** Demographic, Clinical, and Pathological Characteristics of included 138 patients with recurrent RLS

Characteristics	Value (percentage or interquartile range)
Age at surgery (years)	53 (44–61)
Gender	
Male	77 (55.8%)
Female	61 (44.2%)
ASA score (points)	2 (2–3)
Surgery times (times)	2 (2–3)
Tumor growth rate (cm/month)	1.28 (0.63–2.50)
Invaded vessel	
No	76 (55.1%)
Yes	62 (44.9%)
Resection methods	
R0 (Complete resection)	55 (39.9%)
R1 (Positive margin)	58 (42.0%)
R2 (Palliative resection)	25 (18.1%)
Tumor number	
Single	25 (18.1%)
Multifocal	113 (81.9%)
Tumor size (cm)	18 (12–23)
Completeness of tumor capsule	
Complete	73 (52.9%)
Incomplete	65 (47.1%)
Differentiation change	
No	69 (50.0%)
Yes	69 (50.0%)
Combined organ resection	
No	50 (36.2%)
Yes	88 (63.8%)
Recurrence patterns	
DR	63 (45.7%)
LR	75 (54.3%)
Pathological subtypes	
WDL	45 (32.6%)
MLS	35 (25.4%)
PLS	20 (14.5%)
DDL	38 (27.5%)
Pathological classification	
DDL	38 (27.5%)
Non-DDL	100 (72.5%)

*DR* Distant recurrence, *LR* Local recurrence, *RLS* Retroperitoneal liposarcoma, *WDL* Well-differentiated liposarcoma, *DDL* Dedifferentiated liposarcoma, *MLS* Myxoid cell liposarcoma, *PLS* Pleomorphic liposarcoma, *ASA* American Society of Anesthesiologists. Categorical data are expressed as frequencies (percentages) and continuous variables are expressed as the median (Q1-Q3)

### Overall survival and recurrence-free survival

**Fig. 3:**
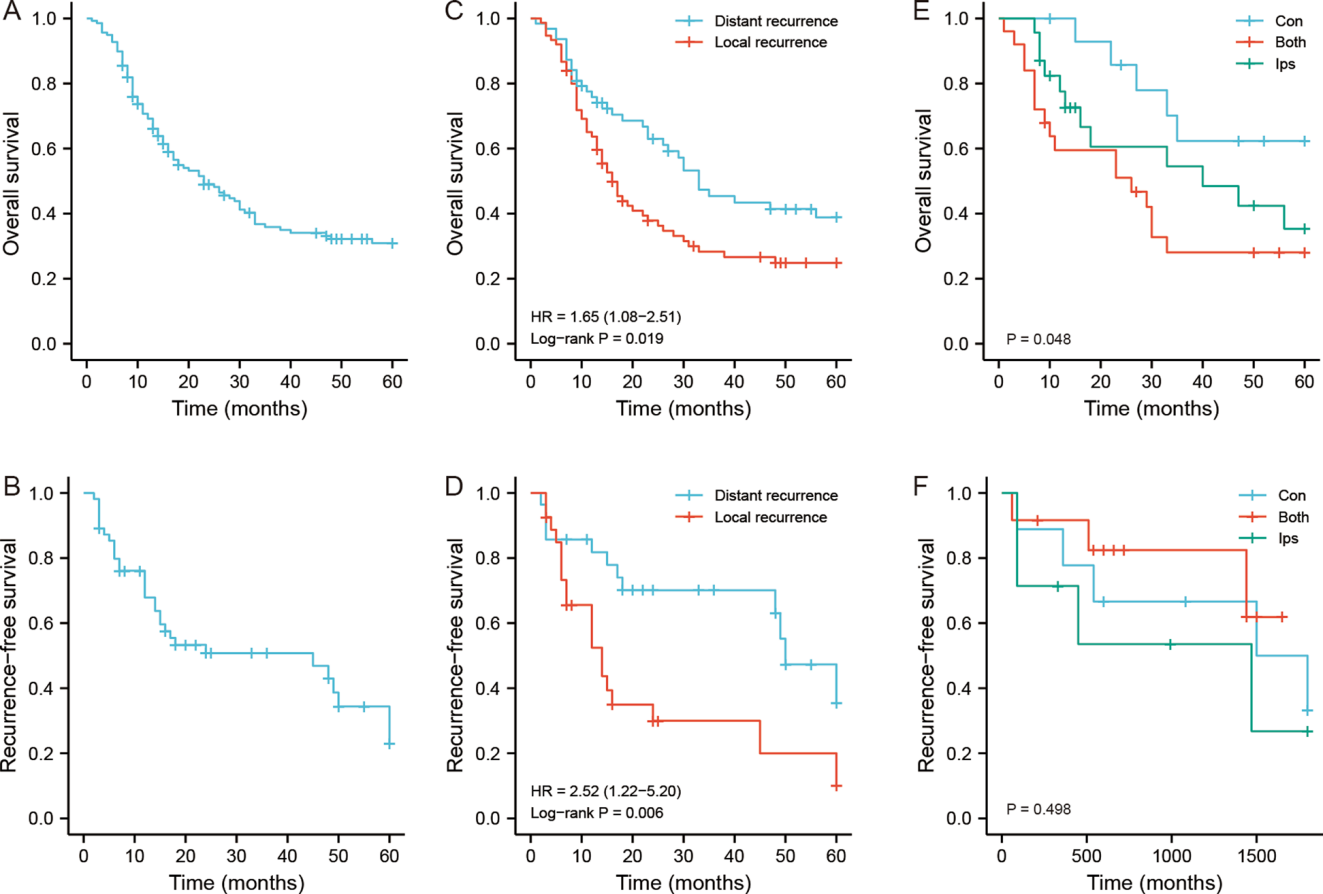
The OS and RFS analysis of patients with recurrent RLS. **A** The OS analysis of all 138 recurrent RLS patients included in this study. **B** The RFS analysis of all 55 patients who received R0 resection. **C** The OS analysis of 138 recurrent RLS patients with two recurrence patterns. D The RFS analysis of 55 recurrent RLS patients who received R0 resection in the two recurrence patterns. **E** The OS analysis of patients with three specific DR modes. **F** The RFS analysis of patients with three specific DR modes. OS: overall survival; RFS: recurrence-free survival; RLS: retroperitoneal liposarcoma; DR: distant recurrence

The median survival time for all patients was about 23 months in this study. The 1-, 3-, and 5-year OS rates were 70.7%, 35.9%, and 30.9%, respectively (Fig. 3A). We further conducted univariate and multivariate Cox analysis to explore the clinicopathologic variables associated with 5-year OS (Table 2). On univariate analysis, the clinicopathologic factors that significantly associated with OS were age at surgery, tumor growth rate, resection methods, status of pathological differentiation, recurrence patterns, pathological subtypes and pathological classification (each *P* < 0.05). These factors were enrolled into multivariate analysis. The results revealed that resection methods, status of pathological differentiation, pathological subtypes and recurrence patterns were independent risk factors for OS (each *P* < 0.05).

**Table 2:** Univariate and multivariate analysis of clinicopathologic variables associated with 5-year OS

Characteristics	Total (N)	Univariate analysis		Multivariate analysis	
		HR (95% CI)	*P* value	HR (95% CI)	*P* value
Age at surgery (years)	138				
≤ 53	74	Reference			
> 53	64	1.565 (1.026–2.386)	**0.038**	1.447 (0.934—2.244)	0.098
Gender	138				
Male	77	Reference			
Female	61	1.119 (0.733–1.708)	0.602		
ASA score (points)	138				
≤ 2	102	Reference			
> 2	36	1.350 (0.843–2.162)	0.212		
Surgery times (times)	138				
2–3	105	Reference			
> 3	33	1.429 (0.887–2.303)	0.143		
Tumor growth rate (cm/month)	138				
≤ 1.28	69	Reference			
> 1.28	69	1.674 (1.096–2.558)	**0.017**	1.270 (0.791—2.039)	0.323
Invaded vessel	138				
No	76	Reference			
Yes	62	1.340 (0.880–2.041)	0.172		
Resection methods	138				
R0/R1 (Grossly complete resection)	113	Reference			
R2 (Palliative resection)	25	3.085 (1.875—5.075)	** < 0.001**	2.685 (1.578—4.571)	** < 0.001**
Tumor number	138				
Single	25	Reference			
Multifocal	113	1.185 (0.679–2.067)	0.551		
Tumor size (cm)	138				
≤ 18	72	Reference			
> 18	66	1.795 (1.176–2.741)	**0.007**	1.578 (1.000—2.491)	**0.050**
Completeness of tumor capsule	138				
Complete	73	Reference			
Incomplete	65	1.307 (0.857–1.993)	0.213		
Status of pathological differentiation	138				
Consistent	69	Reference			
Change	69	1.596 (1.044–2.438)	**0.031**	1.694 (1.069—2.685)	**0.025**
Combined organ resection	138				
No	50	Reference			
Yes	88	1.148 (0.738–1.787)	0.540		
Recurrence patterns	138				
DR	63	Reference			
LR	75	1.665 (1.080–2.567)	**0.021**	1.934 (1.212—3.085)	**0.006**
Pathological subtypes	138				
WDL	45	Reference			
MLS	35	1.855 (1.032–3.335)	**0.039**	2.054 (1.126—3.746)	**0.019**
PLS	20	1.635 (0.848–3.153)	0.142	1.537 (0.765—3.087)	0.228
DDL	38	2.403 (1.362–4.241)	**0.002**	2.180 (1.209—3.930)	**0.010**
Pathological classification	138				
DDL	38	Reference			
Non-DDL	100	0.575 (0.363–0.910)	**0.018**		

*DR* Distant recurrence, *LR* Local recurrence, *OS* Overall survival; *RLS* Retroperitoneal liposarcoma, *HR* Hazard ratio, *CI* Confidence interval, *WDL* Well-differentiated liposarcoma, *DDL* Dedifferentiated liposarcoma, *MLS* Myxoid cell liposarcoma, *PLS* Pleomorphic liposarcoma, *ASA* American Society of Anesthesiologists. The cutoff value was the median value of variable. Bold *P* value refers to *P* < 0.05

We analyzed the recurrence outcomes of patients who underwent complete resection of tumors. The 1-, 3-, and 5-year RFS rates of the 55 patients were 76.1%, 50.8%, and 34.4%, respectively (Fig. 3B). Among the 55 patients, 31 patients experienced recurrence within 5 years. We further explored the risk factors associated with RFS by univariate and multivariate analyses. The results illustrated that pathological differentiation status, pathological subtypes and recurrence patterns were the significant factors associated with RFS (each *P* < 0.05) (Table 3).

**Table 3:** Univariate and multivariate analysis of clinicopathologic variables associated with 5-year RFS in patients with R0 resection

Characteristics	Total (N)	Univariate analysis		Multivariate analysis	
		HR (95% CI)	*P* value	HR (95% CI)	*P* value
Age at surgery (years)	55				
≤ 52	28	Reference			
> 52	27	1.163 (0.573–2.362)	0.676		
Gender	55				
Male	32	Reference			
Female	23	1.057 (0.520–2.149)	0.879		
ASA score (points)	55				
≤ 2	43	Reference			
> 2	12	1.965 (0.867–4.452)	0.106		
Surgery times (times)	55				
2–3	40	Reference			
> 3	15	0.914 (0.393–2.128)	0.835		
Tumor growth rate (cm/month)	55				
≤ 1.22	28	Reference			
> 1.22	27	1.091 (0.536–2.220)	0.810		
Invaded vessel	55				
No	31	Reference			
Yes	24	1.426 (0.698–2.911)	0.330		
Tumor number	55				
Single	9	Reference			
Multifocal	46	0.913 (0.350–2.383)	0.852		
Tumor size (cm)	55				
≤ 18	29	Reference			
> 18	26	0.652 (0.311–1.366)	0.257		
Completeness of Tumor capsule	55				
Complete	37	Reference			
Incomplete	18	1.089 (0.513–2.313)	0.825		
Status of pathological differentiation	55				
Consistent	29	Reference			
Change	26	0.384 (0.176–0.840)	**0.017**	0.443 (0.200–0.983)	**0.045**
Combined organ resection	55				
No	17	Reference			
Yes	38	1.090 (0.496–2.396)	0.830		
Recurrence patterns	55				
DR	28	Reference			
LR	27	2.702 (1.287–5.670)	**0.009**	4.171 (1.757–9.905)	**0.001**
Pathological subtypes	55				
WDL	19	Reference			
MLS	16	1.645 (0.666–4.062)	0.280	2.616 (1.000–6.839)	0.050
PLS	8	1.097 (0.293–4.111)	0.891	1.939 (0.486–7.742)	0.349
DDL	12	3.276 (1.254–8.559)	**0.015**	7.057 (2.318–21.483)	** < 0.001**
Pathological classification	55				
DDL	12	Reference			
Non-DDL	43	0.377 (0.168–0.846)	0.018		

*DR* Distant recurrence, *LR* Local recurrence, *RFS* Recurrence-free survival, *RLS* Retroperitoneal liposarcoma, *HR* Hazard ratio, *CI* Confidence interval, *WDL* Well-differentiated liposarcoma, *DDL* Dedifferentiated liposarcoma, *MLS* Myxoid cell liposarcoma, *PLS* Pleomorphic liposarcoma, *ASA* American Society of Anesthesiologists. The cutoff value was the median value of variable. Bold *P* value refers to *P* < 0.05

**Fig. 4:**
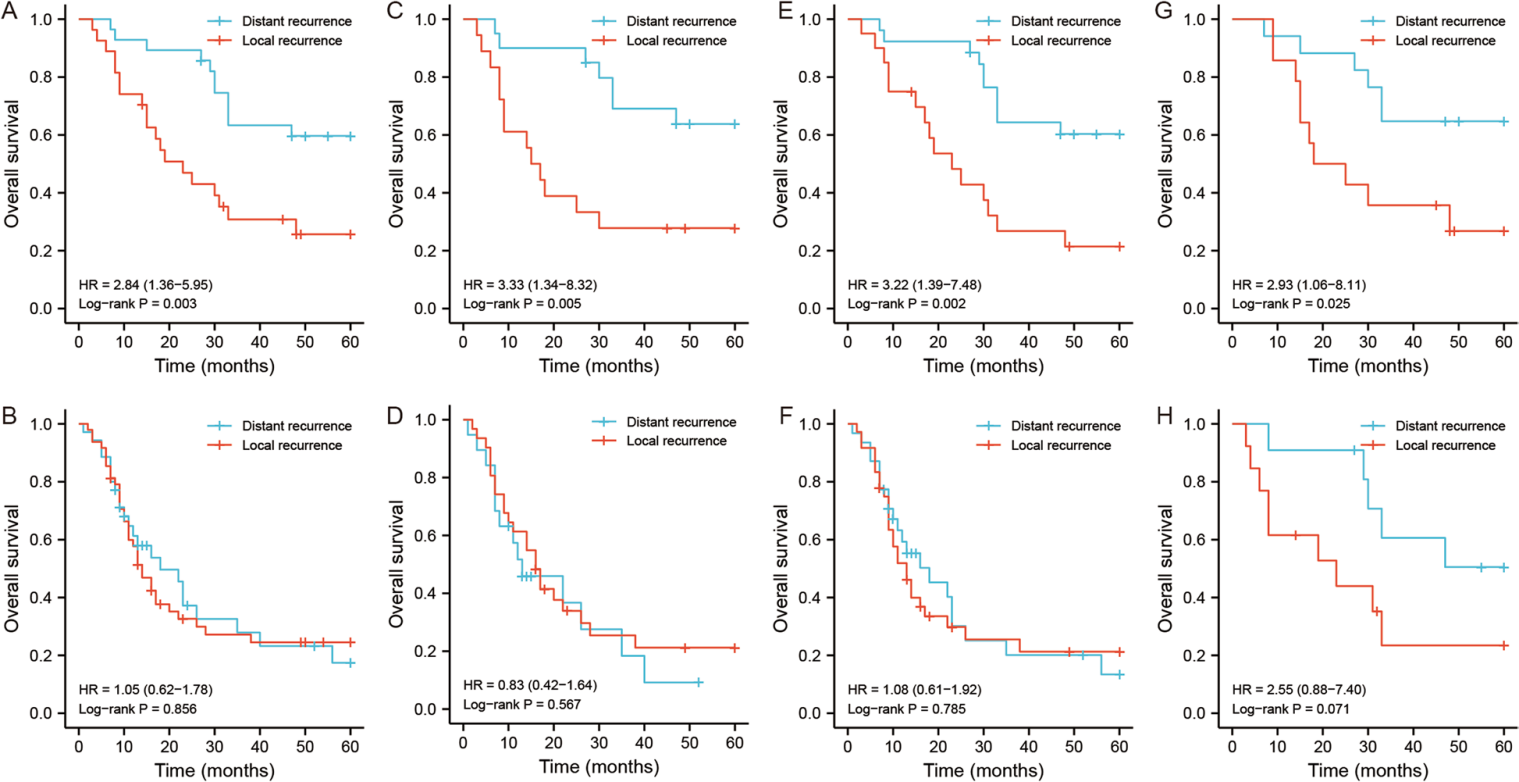
The OS analysis of patients with recurrent RLS in different recurrence patterns. **A** The OS analysis of recurrent RLS patients with DR or LR pattern after R0 resection. **B** The OS analysis of recurrent RLS patients with DR or LR pattern after non-R0 resection. **C** The OS analysis of recurrent RLS patients with DR or LR pattern after combined organ resection. **D** The OS analysis of recurrent RLS patients with DR or LR pattern without combined organ resection. **E** The OS analysis of recurrent RLS patients with multifocal tumors in DR or LR pattern after R0 resection. **F** The OS analysis of recurrent RLS patients with multifocal tumors in DR or LR pattern after non-R0 resection. **G** The OS analysis of recurrent RLS patients with a tumor size less than 18 cm in DR or LR pattern after R0 resection. **H** The OS analysis of recurrent RLS patients with a tumor size greater than 18 cm in DR or LR pattern after R0 resection. OS: overall survival; RFS: recurrence-free survival; RLS: retroperitoneal liposarcoma; DR: distant recurrence; LR, local recurrence

**Fig. 5:**
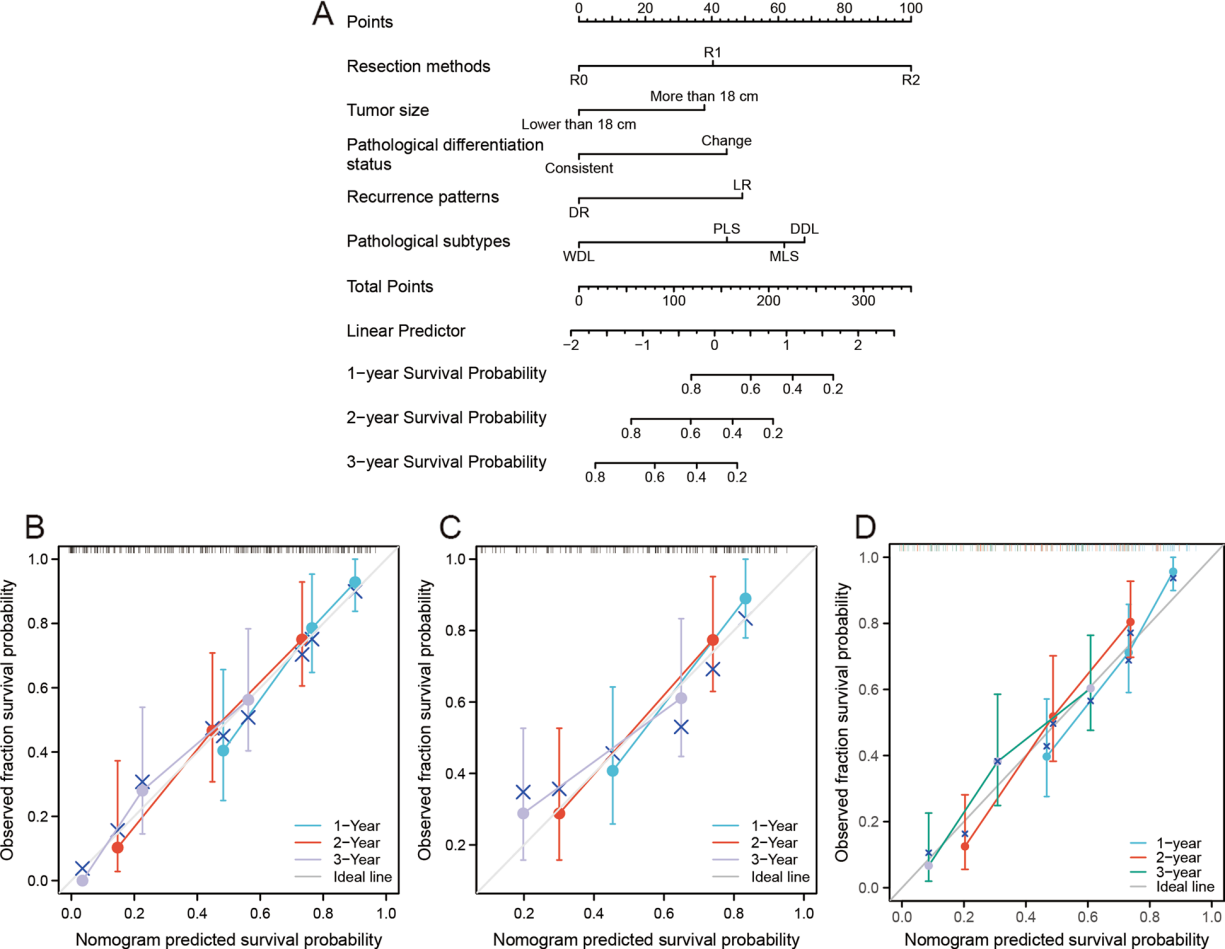
The nomogram model was built to predict the survival status of patients with recurrent RLS. **A** Nomogram for 1-year, 2-year and 3-year overall survival in patients with recurrent RLS. **B** Calibration plots of training set for 1-year, 2-year and 3-year OS in patients with recurrent RLS. **C** Calibration plots of internal validation set for 1-year, 2-year and 3-year OS in patients with recurrent RLS. **D** Calibration plots of external validation set for 1-year, 2-year and 3-year OS in patients with recurrent RLS. The X-axis: bootstrap-predicted survival; the Y-axis: actual outcome. DR, distant recurrence; LR, local recurrence; RLS, retroperitoneal liposarcoma; WDL, well-differentiated liposarcoma; DDL, dedifferentiated liposarcoma; MLS, myxoid cell liposarcoma; PLS, pleomorphic liposarcoma; OS, overall survival. The cutoff value was the median value of the variable (the median value included in the lower side; the 5-year OS or RFS was too low to be displayed in the nomogram model)

The Kaplan‒Meier curves showed that patients with DR pattern had better OS and RFS (Fig. 3C, D). The subgroup analysis for the three specific modes in the DR pattern showed that tumor relapse at contralateral compartments had better survival, and relapse at ipsilateral compartments had poorer survival (Fig. 3E, F). We further conducted survival analysis to identify surgical differences between DR and LR. The stratified analysis based on resection methods illustrated that R0 resection favored the survival of patients with DR, whereas R1 or R2 resection without difference in survival between DR and LR (Fig. 4A, B). Patients with the DR pattern who underwent combined organ resection also had better survival outcomes than patients with the LR pattern (Fig. 4C, D). The tumor number-based stratification analysis showed that patients with multifocal tumors in the DR group benefited survival from R0 resection (Fig. 4E, F). For patients with tumor sizes greater than 18 cm, the survival outcome seemed to have no difference regardless of the surgical methods. However, when the tumor size was lower than 18 cm, patients with R0 had better survival in the DR pattern (Fig. 4G, H). The tumor size was an important factor for the prognostic prediction of RLS. Therefore, we enrolled it into the predictive model. Based on the results of multivariate analysis and prognostic analysis, we further established a nomogram model to predict the survival status of patients with recurrent RLS. The concordance index (C-index) were 0.743 (0.713–0.774), 0.740 (0.694–0.785) and 0.752(0.698–0.806) in training set, validation set and external set, respectively (Fig. 5A-D).

### Recurrence patterns analysis

The recurrence pattern is an important indicator for the clinical management of patients with this ailment. There were some differences among patients who recurrece with LR or DR pattern. We compared the clinicopathological features between the LR and DR groups. The status of pathological differentiation was a significant factor between DR and LR in the entire cohort (Table 4, *P* < 0.05) but did not differ in the RFS subgroup (Table 5, *P* > 0.05). The results showed that patients with DR pattern usually had multifocal tumors, and they were prone to experience multiple recurrence (more than 3 times) and change of pathological differentiation (Table 4, DR 69.8% vs. LR 33.3%, *P* < 0.05). Patients with the DR pattern tended to have a lower incidence of death and recurrence in the study (Table 4, *P* < 0.05).

**Table 4:** Clinicopathological features comparison between DR and LR patterns in all patients

Clinicopathological features	DR (*n* = 63)	LR (*n* = 75)	*P* value
Age at surgery (years)			0.447
≤ 53	36 (*n* = 57.1%)	38 (*n* = 50.7%)	
> 53	27 (*n* = 42.9%)	37 (*n* = 49.3%)	
Gender			0.130
Male	23 (*n* = 36.5%)	37 (*n* = 49.3%)	
Female	40 (*n* = 63.5%)	38 (*n* = 50.7%)	
ASA score			0.542
≤ 2	45 (*n* = 71.4%)	57 (*n* = 76.0%)	
> 2	18 (*n* = 28.6%)	18 (*n* = 24.0%)	
Surgery times (times)			**0.017**
2–3	42 (*n* = 66.7%)	63 (*n* = 84.0%)	
> 3	21 (*n* = 33.3%)	12 (*n* = 16.0%)	
Tumor growth rate (cm/month)			0.864
≤ 1.28	32 (*n* = 50.8%)	37 (*n* = 49.3%)	
> 1.28	31 (*n* = 49.2%)	38 (*n* = 50.7%)	
Invaded vessel			0.068
Yes	40 (*n* = 63.5%)	36 (*n* = 48.0%)	
No	23 (*n* = 36.5%)	39 (*n* = 52.0%)	
Resection methods			0.531
R0/R1 (Grossly complete resection)	53 (*n* = 84.1%)	60 (*n* = 80.0%)	
R2 (Palliative resection)	10 (*n* = 15.9%)	15 (*n* = 20.0%)	
Tumor number			**0.016**
Single	6 (*n* = 9.5%)	19 (*n* = 25.3%)	
Multifocal	57 (*n* = 90.5%)	56 (*n* = 74.7%)	
Tumor size (cm)			0.964
≤ 18	33 (*n* = 52.4%)	39 (*n* = 52.0%)	
> 18	30 (*n* = 47.6%)	36 (*n* = 48.0%)	
Completeness of tumor capsule			0.208
Complete	37 (*n* = 58.7%)	36 (*n* = 48.0%)	
Incomplete	26 (*n* = 41.3%)	39 (*n* = 52.0%)	
Pathological subtypes			0.581
WDL	17 (*n* = 27.0%)	28 (*n* = 37.3%)	
MLS	z15 (*n* = 23.8%)	20 (*n* = 26.7%)	
PLS	14 (*n* = 22.2%)	6 (*n* = 8.0%)	
DDL	17 (*n* = 27.0%)	21 (*n* = 28.0%)	
Pathological classification			0.854
DDL	17 (*n* = 27.0%)	21 (*n* = 28.0%)	
Non-DDL	46 (*n* = 73.0%)	54 (*n* = 72.0%)	
Status of pathological differentiation			** < 0.001**
Change	44 (*n* = 69.8%)	25 (*n* = 33.3%)	
Consistent	19 (*n* = 30.2%)	50 (*n* = 66.7%)	
Combined organ resection			0.676
Yes	39 (*n* = 61.9%)	49 (*n* = 65.3%)	
NO	24 (*n* = 38.1%)	26 (*n* = 34.7%)	
Survival status			**0.043**
Death	34 (*n* = 54.0%)	53 (*n* = 70.7%)	
Other	29 (*n* = 46.0%)	22 (*n* = 29.3%)	

*DR* Distant recurrence, *LR* Local recurrence, *RLS* Retroperitoneal liposarcoma, *WDL* Well-differentiated liposarcoma, *DDL* Dedifferentiated liposarcoma, *MLS* Myxoid cell liposarcoma, *PLS* Pleomorphic liposarcoma, *ASA* American Society of Anesthesiologists. Categorical data are expressed as frequencies (percentages). The cutoff value was the median value of variable. Bold *P* value refers to *P* < 0.05

**Table 5:** Clinicopathological features comparison between DR and LR patterns in patients with R0 resection

Clinicopathological features	DR (*n* = 28)	LR (*n* = 27)	*P* value
Age at surgery (years)			0.504
≤ 52	16 (*n* = 57.1%)	13 (*n* = 48.1%)	
> 52	12 (*n* = 42.9%)	14 (*n* = 51.9%)	
Gender			0.139
Male	19 (*n* = 67.9%)	13 (*n* = 48.1%)	
Female	9 (*n* = 32.1%)	14 (*n* = 51.9%)	
ASA score			0.943
≤ 2	22 (*n* = 78.6%)	21 (*n* = 48.1%)	
> 2	6 (*n* = 21.4%)	6 (*n* = 48.1%)	
Surgery times			**0.042**
2–3	17 (*n* = 60.7%)	23 (*n* = 85.2%)	
> 3	11 (*n* = 39.3%)	4 (*n* = 14.8%)	
Tumor growth rate (cm/month)			0.498
≤ 1.22	13 (*n* = 46.4%)	15 (*n* = 55.6%)	
> 1.22	15 (*n* = 53.6%)	12 (*n* = 44.4%)	
Invaded vessel			0.671
Yes	15 (*n* = 53.6%)	16 (*n* = 59.3%)	
No	13 (*n* = 46.4%)	11 (*n* = 40.7%)	
Tumor number			0.129
Single	2 (*n* = 7.1%)	7 (*n* = 25.9%)	
Multifocal	26 (*n* = 92.9%)	20 (*n* = 74.1%)	
Tumor size (cm)			0.508
≤ 18	17 (*n* = 60.7%)	14 (*n* = 51.9%)	
> 18	11 (*n* = 39.3%)	13 (*n* = 48.1%)	
Completeness of tumor capsule			0.925
Complete	19 (*n* = 67.9%)	18 (*n* = 66.7%)	
Incomplete	9 (*n* = 32.1%)	9 (*n* = 33.3%)	
Pathological subtypes			0.468
WDL	8 (*n* = 28.6%)	11 (*n* = 40.7%)	
MLS	7 (*n* = 25.0%)	9 (*n* = 33.3%)	
PLS	5 (*n* = 17.8%)	3 (*n* = 11.1%)	
DDL	8 (*n* = 28.6%)	4 (*n* = 14.8%)	
Pathological classification			0.217
DDL	8 (*n* = 28.6%)	4 (*n* = 14.8%)	
Non-DDL	20 (*n* = 71.4%)	23 (*n* = 85.2%)	
Status of pathological differentiation			0.135
Change	16 (*n* = 57.1%)	10 (*n* = 37.0%)	
Consistent	12 (*n* = 42.9%)	17 (*n* = 63.0%)	
Combined organ resection			0.931
Yes	20 (*n* = 71.4%)	19 (*n* = 70.4%)	
NO	8 (*n* = 28.6%)	8 (*n* = 29.6%)	
Recurrence status			
Recurrence	12 (*n* = 42.9%)	19 (*n* = 70.4%)	**0.04**
Other	16 (*n* = 57.1%)	8 (*n* = 29.6%)	

*DR* Distant recurrence, *LR* Local recurrence, *RLS* Retroperitoneal liposarcoma, *WDL* Well-differentiated liposarcoma, *DDL* Dedifferentiated liposarcoma, *MLS* Myxoid cell liposarcoma, *PLS* Pleomorphic liposarcoma, *ASA* American Society of Anesthesiologists. The cutoff value was the median value of variable. Categorical data are expressed as frequencies (percentages). Bold *P* value refers to *P* < 0.05

**Fig. 6:**
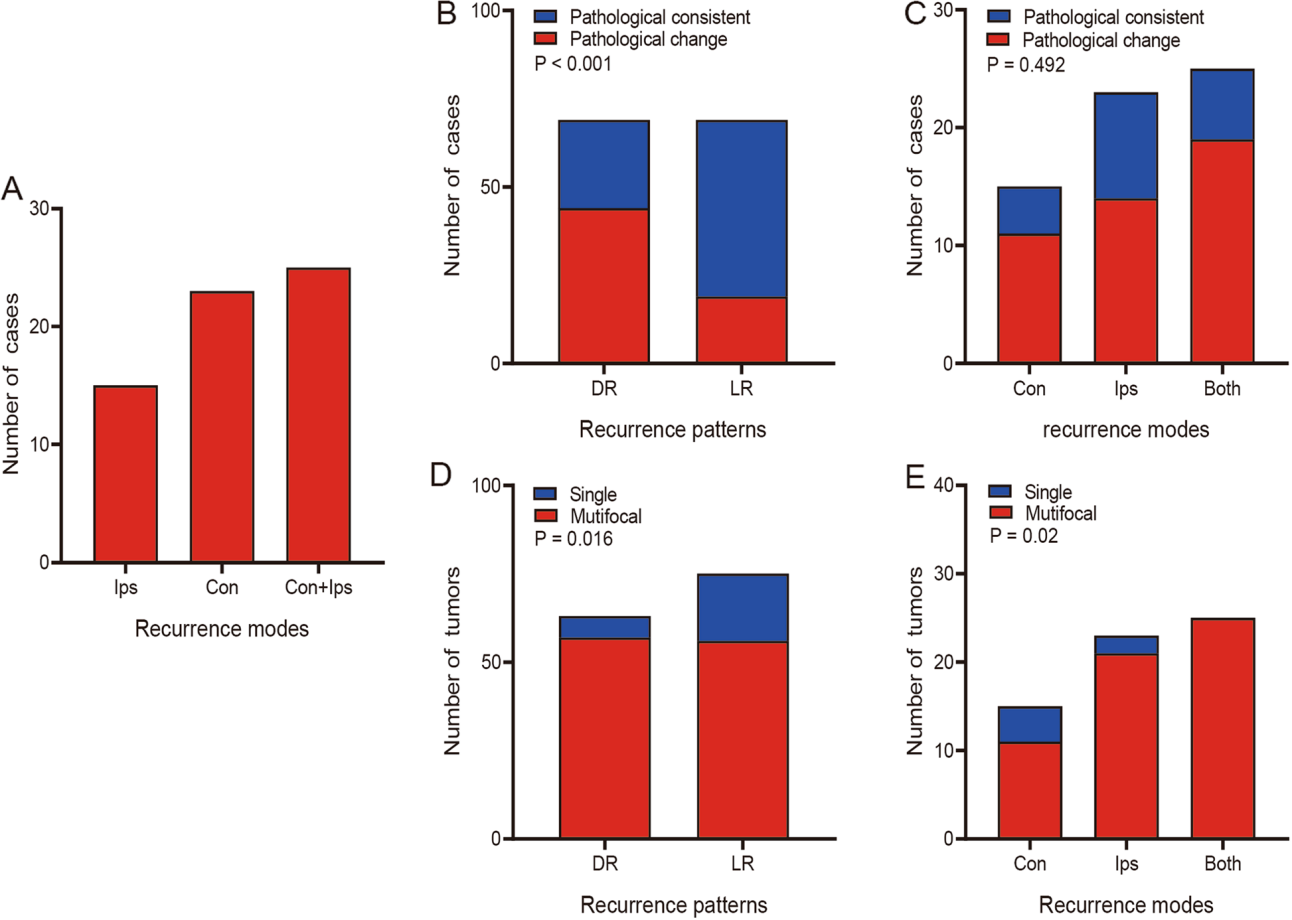
Histogram showing the number of recurrent RLS cases with pathological changes or multifocal tumors in different recurrence patterns. **A** The total number of cases in three specific DR moeds. **B** The number of cases with pathological changes in DR and LR patterns. **C** The number of cases with pathological changes among the three specific DR modes. **D** The number of cases with multifocal tumors in DR and LR patterns. **E** The number of cases with multifocal tumors among the three specific DR modes. RLS: retroperitoneal liposarcoma. DR: distant recurrence. LR: local recurrence

We further divided the DR pattern into three specific modes according to the alteration of tumor location during relapse. Recurrence occurred at ipsilateral compartments, contralateral compartments or both, with 15 cases, 23 cases and 25 cases, respectively (Fig. 6A). The proportion of changes in pathological differentiation between DR and LR was differently (Fig. 6B), but there were no significant differences among three specific modes of DR (Fig. 6C). However, the tumor number illustrated a significant impact on three specific recurrence modes. There were significant differences in tumor numbers between DR and LR. Multifocal tumors were more frequent in the DR group (Fig. 6D). Among three specific modes of DR, the number of tumor also showed an obvious difference (Fig. 6E).

## Discussion

RLS is a very rare disease, and the most effective treatment for those patients is surgery [16]. Unfortunately, the high probability of recurrence after surgery becomes the treatment dilemma. Therefore, it is urgent to explore factors associated with prognosis to achieve an accurate recurrence risk assessment for patients with recurrent RLS.

In this study, we attempted to investigate the baseline clinicopathological characteristics of recurrent RLS patients and explore the independent prognostic factors that are correlated with OS or RFS. The multivariate analysis showed that resection methods, tumor size, pathological subtypes, status of pathological differentiation, and recurrence patterns were significant prognostic factors (Table 1). Some of the results are consistent with previous studies. The resection method is an very important prognostic factor for RLS and has been widely accepted. R0 resection usually promotes the prognosis of patients with recurrent RLS [7, 11]. Tumor size is another predictive factor of recurrent RLS. In the cohort reported by YI-XI WU et al., patients with tumor sizes larger than 20 cm had poor prognoses [17]. James et al. found that tumor size and tumor growth rate independently influenced the development of a second local recurrence [3]. Likewise, pathology also plays an important role during the recurrence of RLS. The research finished by Sanjay et al. proved that pathologic subtypes of recurrent RLS influence patient prognosis. They found that DDL with the potential of locally aggressive and distant metastasis [12]. Interestingly, the recurrence of RLS was usually accompanied by a change of pathological differentiation. Carolyn et al. found that changes in pathological differentiation can impact the recurrence of RLS. When WDL recurs as DDL, survival and local recurrence are both impacted [18, 19]. Our study also found that changes in pathological differentiation were an independent risk factor of OS for patients with recurrent RLS.

The recurrence of RLS is a common phenomenon, and the recurrence pattern remain an very important factor for the prognosis of patients. Local recurrence influences the prognosis of patients with recurrent RLS that have been accepted widely. However, consensus for the definition of local range remain unclear. Some studies defined that RLS recurrences at the retroperitoneal space were local recurrence [8, 12]. Carolyn et al. deemed local recurrence as any retroperitoneal or intraabdominal (regional) recurrence within the peritoneal cavity and pelvis [18]. Likewise, in recurrent RLS, distant recurrence has been seen as a predictor of poor prognosis [20, 21]. William et al. defined a tumor that relapsed at another compartment of the retroperitoneum as “outside field recurrence”, replacing the “distant” expression [11]. Some studies have directly linked distant organ metastases with distant recurrence [7, 22], Whereas, some studies have even classified organ metastasis as distant metastasis [23]. It can be seen that the definition of local or distant recurrence is controversial in recurrent RLS. The findings from these studies did not clarify the status of recurrent RLS. In our study, we first defined an explicit anatomical range of recurrence in the retroperitoneum and then compared the differences between LR pattern and DR pattern.

We divided recurrent RLS into DR and LR patterns according to the alteration of tumor location during relapse. The DR pattern indicates that the center location of tumors shifted into other anatomical compartments after surgery. The LR pattern is a locoregional problem. The tumor location of RLS with LR pattern did not change in the next recurrence. In the series reported by William et al. [11], the rates of locoregional recurrence of RLS were threefold higher than those of other tumors in the retroperitoneum. The locoregional recurrence of RLS is a severe obstacle for radical cure of RLS. However, there are lack of high-quality studies that thoroughly explored the clinical characteristics of distant recurrence in recurrent RLS, and little is known about how the DR pattern influences the prognosis of RLS patients.

To explore the distant recurrence mode of RLS in the retroperitoneum, we excluded cases with distant organ metastases and comprehensively compared the differences between LR and LR patterns. We found that OS and RFS were distinctly differ in the two patterns. Patients with the DR pattern had a better prognosis than those with the LR pattern (Fig. 3C, D). In addition, patients with the DR pattern were prone to have multifocal tumors and change in pathological differentiation (Table 3). These manifestations may influence the survival outcomes. A large number of studies have shown that primary RLS has a better prognosis than recurrent RLS [7, 12, 24]. In the study conducted by William et al. [11], the survival status of recurrent patients with unifocal or multifocal was influenced by pathology. WDL Patients could benefit survival from unifocal status during recurrence. However, it is insignificance in patients with DDL. The specific feature is different from primary or de novo RLS. In terms of the tumor numbers of recurrent RLS, patients with DR pattern usually have multifocal tumors. Some tumors might be new-onset neoplasm in the distant retroperitoneum. Those tumors grow like primary tumors with lower aggressive biology. Therefore, the survival status of patients with the DR pattern was more inclined to primary RLS. In addition, the change in pathological differentiation was another evidence to support this hypothesis. The previous researches concluded that pathological differentiation change from WDL to DDL or DDL to WDL may influence the prognosis of RLS [18, 19]. Some studies have demonstrated that the four subtypes of RLS have specific typical gene aberrations and biological features, and their biological behaviors and clinical characteristics are dissimilar [25]. Thus, the change of pathological differentiation during relapse deserve increased attention. It is possible that a new-onset tumor grew with a new pathological subtype in the distant retroperitoneum and exhibited pathological differentiation in the next recurrence, which inevitably influenced the survival status. The proportion of changes in tumor number and pathological differentiation was lower in patients with LR pattern. Tumor recurrence with this pattern maintained a stable original malignancy or even became more aggressive in the retroperitoneum [24]. Therefore, patients recurrence with the LR pattern may have a poor prognosis.

We explored the difference in survival status between DR and LR patterns. Patients with the DR pattern had better OS, and patients with the LR pattern had shorter RFS. In other words, LR has a higher recurrence rate and shorter recurrence interval, which provides evidence to back up the belief that different management strategies are required for DR and LR. Patients with LR pattern should receive a shorter follow-up interval during clinical management. The Kaplan‒Meier curves showed that all patients who experienced non-R0 resection had poor survival in both DR and LR. However, patients with the DR pattern benefited more from R0 resection than those with the LR pattern (Fig. 4A, B). Previous studies have proven the significance of aggressive surgical treatment for recurrent RLS [15, 26]. In addition, combined organ resection also had similar clinical implications in survival. Patients with the DR pattern had better survival outcomes when conducted a combined organ resection. Tumor size is an important indicator of survival in recurrent RLS [5, 16]. Patients with tumor greater than 18 cm had a poor prognosis regardless of the resection methods. However, patients could benefit more from R0 resection if the tumor size less than 18 cm. Likewise, patients with DR pattern recurrence with multifocal tumors also benefited from R0 resection (Fig. 4G, H). So, to sum up, we suppose that aggressive surgery may be an option for patients with DR pattern in recurrent RLS [15].

We further analyzed the prognosis of three specific modes of patients with DR pattern. The Kaplan‒Meier curves showed that patients recurrence with tumor location on different sides of retroperitoneum had different prognosis. Patients reccurrence at the contralateral retroperitoneum had a better prognosis. However, reccurrence at ipsilateral or two sides of retroperitoneum simultaneously had a poorer prognosis. Those patients reccurrence at ipsilateral compartments had the poorest prognosis (Fig. 3E, F). We speculated that the phenomenon was associated with tumor numbers and pathological differentiation status. The tumor recurrence at contralateral compartments of retroperitoneum may originates from a new tumor onset point, which has different pathological and biological characteristics. However, it was not as significant as the comparison between the LR and DR groups. We assumed that multifocal tumors and the change of pathological differentiation were so frequent among the three modes.

Regarding the impact of recurrence patterns in recurrent RLS, few studies showed the clinical importance of recurrence pattern in the retroperitoneum. This study concluded the significant differences of prognosis between DR pattern and LR pattern in patients with recurrent RLS. RLS Patients recurrence at the distant anatomical regions of retroperitoneum need a new understanding. During long-term clinical practice, we found that some tumors recurrence with DR pattern may originate from new-onset points, resemble a primary tumor. Those tumors were latent or microscopic status at the distant retroperitoneal regions, which cannot be perceived during the last surgical procedure and subsequently lead to the next recurrence. Multiple different gene aberrations were detected in patients with recurrent RLS [4], which was the evidence that a patient may have multiple tumor growth points in the retroperitoneum concurrently. Hence that, we can found that patients with DR pattern were more likely to have multifocal tumors and changes in pathological differentiation during the process of recurrence. However, tumors with LR pattern usually originate from the same tumor growth points or residual tumor cells, which might be true recurrence.

Given the rarity of recurrent RLS, the prognostic values of clinicopathological features in those patients remain unclear. There are lack of clinical models to predict the survival of recurrent RLS. In this study, we built a high efficient nomogram model to predict the survival of patients with recurrent RLS. The nomogram can provide a visual interface to aid in calculating the predicted probability that a patient will achieve a particular clinical endpoint [27]. The previous studies in primary RLS revealed that age, gender, clinical manifestations, pathology and resection method were important survival predictors for patients with primary RLS [2, 27, 28]. This study enrolled recurrence pattern and the status of pathological differentiation in the nomogram model for predicting survival of recurrent RLS. Actually, the adjuvant therapy especially radiotherapy is an important method to decrease and predict the probability of local recurrence. However, the smaller data volume of the study limited the case inclusion in those patients.

### Study limitations

Our study has several limitations. First, this study involved only a single institution, the number of cases was limited, and adjuvant therapies and distant metastases were unable to be further analyzed. Second, the long-term survival and disease-specific death for recurrent RLS should be evaluated. Third, our cases were retrospective, mainly based on our institution’s medical records, and lacked prospective data. Fourth, the data for some factors, such as tumor necrosis and the mitotic count, were missed, making pathological grading difficult.

## Conclusion

The multivariate Cox analysis revealed that resection method, tumor size, status of pathological differentiation, pathological subtypes and recurrence patterns were independent prognostic factors for OS or RFS. Patients with DR pattern were prone to have multifocal tumors and changes in pathological differentiation. Those patients may benefit more from aggressive combined resection. Patients with the LR pattern showed shorter OS and RFS, who should receive a shorter follow-up interval after surgery. Classifying recurrent RLS as LR and DR patterns in the retroperitoneum contributes to a better understanding of the recurrence mechanisms of RLS, and provides guidance for individualized clinical management.

### Supplementary Information

**Additional file 1:** **Supplementary Figure 1. **The changes of pathological differentiation among subtypes of RLS in twice of continuous recurrences. (A) The changes of pathological differentiation in all enrolled cases. (B) The changes of pathological differentiation in patients with DR pattern. (C) The changes of pathological differentiation in patients with LR pattern. RLS, retroperitoneal liposarcoma; DR: distant recurrence. LR: local recurrence. RLS: retroperitoneal liposarcoma; WDL: well-differentiated liposarcoma; DDL: dedifferentiated liposarcoma; MLS: myxoid cell liposarcoma; PLS, pleomorphic liposarcoma.

## Data Availability

Data analyzed in the study are available upon request pending application and authority approval. Requests to access the datasets should be directed to BW, PLAweibo@163.com.
