# Supplementary material for: Predictors and outcomes of recurrent retroperitoneal liposarcoma: new insights into its recurrence patterns
Source: BMC Cancer. 2023 Nov 8;23:1076. doi: 10.1186/s12885-023-11586-8 (PMC10631151; doi:10.1186/s12885-023-11586-8)
Supplement: Supplementary file 1 — Additional file 1: Supplementary Figure 1. The changes of pathological differentiation among subtypes of RLS in twice of continuous recurrences. (A) The changes of pathological differentiation in all enrolled cases. (B) The changes of pathological differentiation in patients with DR pattern. (C) The changes of pathological differentiation in patients with LR pattern. RLS, retroperitoneal liposarcoma; DR: distant recurrence. LR: local recurrence. RLS: retroperitoneal liposarcoma; WDL: well-differentiated liposarcoma; DDL: dedifferentiated liposarcoma; MLS: myxoid cell liposarcoma; PLS, pleomorphic liposarcoma. [file 12885_2023_11586_MOESM1_ESM.zip › Supplementary legends.docx]

**Supplementary Figure 1.** The changes of pathological differentiation among subtypes of RLS in twice of continuous recurrences. (A) The changes of pathological differentiation in all enrolled cases. (B) The changes of pathological differentiation in patients with DR pattern. (C) The changes of pathological differentiation in patients with LR pattern. RLS, retroperitoneal liposarcoma; DR: distant recurrence. LR: local recurrence. RLS: retroperitoneal liposarcoma; WDL: well-differentiated liposarcoma; DDL: dedifferentiated liposarcoma; MLS: myxoid cell liposarcoma; PLS, pleomorphic liposarcoma.
